# Performance Analysis of Polymer Electrolyte Membrane Water Electrolyzer Using OpenFOAM^®^: Two-Phase Flow Regime, Electrochemical Model

**DOI:** 10.3390/membranes10120441

**Published:** 2020-12-18

**Authors:** Kyu Heon Rho, Youngseung Na, Taewook Ha, Dong Kyu Kim

**Affiliations:** 1 School of Mechanical Engineering, Chung-Ang University, Seoul 06974, Korea; aks05041@cau.ac.kr (K.H.R.); htu5797@cau.ac.kr (T.H.); 2 School of Computer Science and Engineering, Chung-Ang University, Seoul 06974, Korea; 3 Department of Mechanical and Information Engineering, University of Seoul, Seoul 02504, Korea; ysna@uos.ac.kr

**Keywords:** polymer electrolyte membrane water electrolyzer, two-phase flow, electrochemical reaction, performance analysis, performance comparison of flow field

## Abstract

In this study, an electrochemical model was incorporated into a two-phase model using OpenFOAM^®^ (London, United Kingdom) to analyze the two-phase flow and electrochemical behaviors in a polymer electrolyte membrane water electrolyzer. The performances of serpentine and parallel designs are compared. The current density and overpotential distribution are analyzed, and the volume fractions of oxygen and hydrogen velocity are studied to verify their influence on the current density. The current density decreases sharply when oxygen accumulates in the porous transport layer. Therefore, the current density increased sharply by 3000 A/m^2^ at an operating current density of 10,000 A/m^2^. Maldistribution of the overpotential is also observed. Second, we analyze the behaviors according to the current density. At a low current density, most of the oxygen flows out of the electrolyzer. Therefore, the decrease in performance is low. However, the current density is maldistributed when it is high, which results in decreased performance. The current density increases abruptly by 12,000 A/m^2^. Finally, the performances of the parallel and serpentine channels are analyzed. At a high current density, the performance of the serpentine channel is higher than that of the parallel channel by 0.016 V.

## 1. Introduction

With the increased importance of green energy, many researchers are interested in the use of hydrogen as an energy transport material [1,2]. Due to the fact that the present price of hydrogen in the Republic of Korea is USD 7.17/kg, it is not economical to convert the country’s energy base to hydrogen [3]. Therefore, it is necessary to reduce the price of hydrogen through a new technical development. The polymer electrolyte membrane water electrolyzer (PEMWE) has attracted attention as a potential hydrogen production tool. The PEMWE can produce hydrogen with high purity (99%) [4] and operate at high current densities [5]. Therefore, it is suitable for producing large amounts of hydrogen [6]. However, when the current density becomes excessively high, the efficiency of the PEMWE can decrease, since the generated oxygen bubbles disturb the contact between the reactant and catalyst [7,8]. Hence, it is necessary to analyze the phenomenon in the PEMWE and identify a suitable flow field to eliminate the generated oxygen effectively, as well as to uniformly distribute the reactant.

Many researchers have attempted to experimentally analyze the performances of the PEMWE and PEMWE systems. A few researchers performed experiments to examine the reactivity of the water decomposition reaction for different catalysts. They observed the existence of an exchange current density of the hydrogen reduction reaction (HRR) and oxygen oxidation reaction (OOR). For a platinum catalyst, the exchange current densities for the HRR and OOR are reported to be 1.61 × 10^−1^–1.61 × 10^−2^ A/m^2^ [9,10] and 1.61 × 10^−9^–1.61 × 10^−6^ A/m^2^ [10,11], respectively. When an iridium—platinum catalyst was used for the reaction, the exchange current density of the ORR increased to 1.61 × 10^−4^ A/m^2^ [10,12].

Simultaneously, many researchers have attempted to analyze the influence of two-phase flow on the performance of the PEMWE. A few researchers measured the volume fraction and pressure loss of the water-air flow in capillary tubes [13]. They proposed an empirical equation to calculate the pressure loss using a capillary tube diameter. Other researchers conducted experiments to analyze the two-phase flow in a porous transport layer (PTL) [14]. They verified the relationship between the electrode porosity, bubble diameter of gas, and performance of the PEMWE. The performance increased with the increasing electrode porosity. However, there was no significant effect when the porosity exceeded 0.5. Other researchers have used the transparent cell to observe the two-phase flow in an electrolyzer [15]. They verified that bubbles were converted into slug flow when the superficial gas velocity exceeded 8 × 10^−2^ m/s for an operating current density of 700 A/m^2^. However, the existing studies observed only the two-phase flow without considering the electrochemical reaction, and it was also insufficient for predicting the performance using the flow field design of the PEMWE.

Numerical analyses have been performed to analyze the two-phase flow of various channel designs. A few researchers observed that the pressure drop increased with the increasing oxygen generation in a parallel flow field [16]. In this study, they assumed that oxygen gas was produced uniformly at the interface between the PTL and membrane, to replicate the electrochemical reaction in the membrane. Other researchers have analyzed the two-phase flow of a circular and interdigitated flow field of the PEMWE to verify the difference in the distribution of the oxygen-water mixture, according to the particle size for this numerical model [17]. They verified that the maximum gas volume fraction increased from 0.4 to 0.45 for a particle diameter of 40 µm. The thermal and electrochemical reactions in the PEMWE have also been analyzed [18]. These studies verified that the local temperature increased by 20.2 °C owing to the oxygen bubble in the PTL at an operating current density of 1500 A/m^2^. Although the relationship between the performance of the PEMWE and two-phase flow have been analyzed in many previous studies, this did not adequately explain the relationship between the two-phase flow in the anode and the electrochemical reaction in the membrane. It is necessary to develop a numerical model to analyze the interaction between the electrochemical and two-phase flow phenomena.

In this study, we developed a transient model of PEMWE to analyze the two-phase flow and electrochemical phenomenon of various channel designs. We applied the Euler-Euler two-phase model and the Butler-Volmer equation as the electrochemical model for the PEMWE. First, the proposed model was validated using experimental data. Then, the current density and overpotential distributions were analyzed to understand the performance of the PEMWE. The current density and overpotential in the membrane were analyzed, and the performance was studied according to the operating current density. Finally, the performances of the serpentine and parallel channels were compared. This study contributes to the development of a numerical model and optimizes the flow field of the PEMWE.

## 2. Methodology 

The PEMWE comprises a membrane electrode assembly (MEA), gasket, bipolar plate, and current collector. The MEA of the PEMWE comprises the PTL for the anode and cathode, a catalyst, and a membrane. Liquid water flows into the anode, and the oxygen-water mixture flows out of it. However, no fluid flows into the cathode, and hydrogen flows out of it. The standard voltage of the reaction is 1.23 V, and this reaction is given by Equation (1) [19]:

(1) $$\left\{ \begin{array}{cccc} H_{2}O\; & \to & \frac{1}{2}O_{2}{+2H}^{+}{+2e}^{-} & \;(\mathrm{anode})\\ {2H}^{+}{+2e}^{-} & \to & H_{2} & \;(\mathrm{cathode}) \end{array}\right.$$

**Figure 1:**
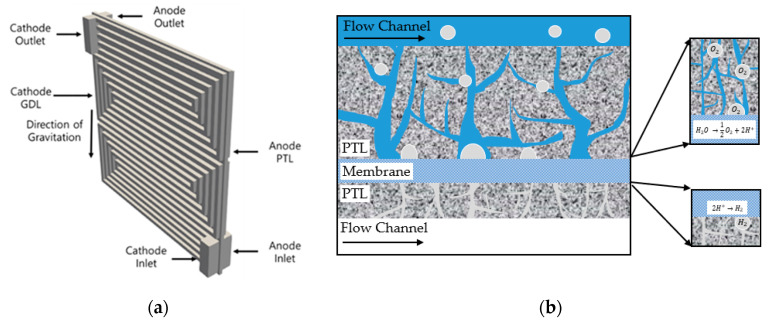
(**a**) Geometry of a five-serpentine flow field. (**b**) The phenomenon with the two-phase flow and electrochemical reactions in the polymer electrolyte membrane water electrolyzer (PEMWE).

The five-serpentine flow field shown in Figure 1a was used to analyze the two-phase flow and electrochemical behavior. The geometric parameters are listed in Table 1. The phenomenon that occurred inside the cell is identical to that shown in Figure 1b. In this study, an electrochemical model was incorporated into two-phase and single-phase models using OpenFOAM^®^ (Open Field Operation and Manipulation, London, United Kingdom) to explain the two-phase and single-phase flows and electrochemical reaction in the PEMWE. In addition, this model was verified using the experimental results.

### 2.1. Electrochemical Model

The Nernst voltage (*E*^0^) depends on the reactant/product activities and the standard potential of the reaction (*E*_0_), as demonstrated in [20]:

(2) $$E^{0}=E_{0}-\frac{RT}{F}\ln\left(\frac{\prod a_{product}}{\prod a_{reactant}}\right)$$

where *R* is the universal gas constant, *T* is the temperature, *F* is Faraday’s constant, and a represents the activity of the reactant and product. The activation overpotential (*η*) of the reaction is computed using the open circuit voltage (OCV, *E*), ohmic loss (*E*_ohm_), and Nernst voltage. It can be expressed as follows:

(3) $$\eta =E-E^{0}-E_{ohm}$$

The Butler-Volmer equation explains the relationship between the activation overpotential and current density of the reaction. It is expressed as follows [21]:

(4) $$\left\{ \begin{array}{l} i_{A}=i_{A0}\left\{\exp\left(\frac{2\alpha_{A}F\eta_{A}}{RT}\right)-\exp\left(-\frac{2\left(1-\alpha_{A}\right)F\eta_{A}}{RT}\right)\right\}\\ i_{C}=i_{C0}\left\{\exp\left(\frac{2\alpha_{C}F\eta_{C}}{RT}\right)-\exp\left(-\frac{2\left(1-\alpha_{C}\right)F\eta_{C}}{RT}\right)\right\} \end{array}\right.$$

where *i_A_* and *i_C_* are the current densities, *i_A_*_0_ and *i_C_*_0_ are the exchange current densities, *η_A_* and *η_C_* are the activation overpotentials, and *α_A_* and *α_C_* are the transfer coefficients for the anode and cathode reactions, respectively. The exchange current densities depend on the temperature [10], as follows:

(5) $$\left\{ \begin{array}{l} i_{A0}=\alpha_{W}\gamma_{m}\exp\left(-\frac{E}{R}\left[\frac{1}{T}-\frac{1}{T_{ref}}\right]\right)i_{A0}^{ref}\\ i_{C0}=\gamma_{m}\exp\left(-\frac{E}{R}\left[\frac{1}{T}-\frac{1}{T_{ref}}\right]\right)i_{C0}^{ref} \end{array}\right.$$

where *i_A_*_0_*^ref^* and *i_C_*_0_*^ref^* are the reference exchange current densities of the anode and cathode reactions, respectively, *γ_m_* is the roughness factor of the electrode, *α_W_* is the volume fraction of water at the interface between the membrane and PTL, *E* is the effective activation energy, and *T_ref_* (=298.15 K) is the reference temperature (listed in Table 2). The reference exchange current density of the oxygen reduction reaction (ORR) is 10^−3^ A/m^2^ for the Ir–Pt catalyst and that of the hydrogen oxidation reaction (HOR) is 1 A/m^2^ for the Pt catalyst. The exchange current density for the reverse reaction is computed by dividing the equilibrium constant of reaction (=6.2). The ohmic loss in the membrane is computed using the current density and area resistance of the membrane. The ohmic loss and area resistance are computed as follows [22]:

(6) $$\left\{ \begin{array}{l} E_{ohm}=ASR_{m}\times i\\ ASR_{m}=\int \frac{1}{\sigma }dz \end{array}\right.$$

where *ASR_m_* and *σ* are the area resistance and conductivity, respectively, of the membrane.

### 2.2. Conservation Equation

Both the liquid water and oxygen gas flow in the anode. Therefore, the Euler-Euler two-phase model is applied to the anode. The continuity equation of the two-phase flow is expressed as follows [24]:

(7) $$\frac{\partial }{\partial t}\alpha_{i}\rho_{i}+\nabla \cdot (\alpha_{i}\rho_{i}U_{i})=0$$

where *ρ_i_* is the density (listed in Table 3), *α_i_* and *U_i_* are the volume fraction and velocity, respectively, of species *i* (water and oxygen), *α_i_* is the ratio of the species volume to the total volume (*V_i_/V_tot_*), and *α_W_* + *α_O_* = 1, since the sum of all the species becomes one. Then, the momentum equation for the liquid water and oxygen is [25]:

(8) $$\nabla \cdot \left(\alpha_{i}\rho_{i}U_{i}U_{i}\right)=-\alpha_{i}\nabla P+\nabla \cdot \left(\alpha_{i}\mu_{eff}\left(\nabla \mu_{k}+{\left(\nabla \mu \right)}^{T}\right)\right)+\alpha_{i}\rho_{i}g+M_{k}$$

where *p* is the pressure, *g* is the acceleration of gravity, *µ_eff_* is the effective viscosity, and *M_k_* is the interfacial momentum exchange term, which is the sum of the forces owing to drag, lift, and turbulent dispersion. The effective viscosity is the sum of the liquid viscosity and turbulent viscosity. It is calculated as follows [25]:

(9) $$\left\{ \begin{array}{l} \mu_{eff}=\mu_{W}+\mu_{t}\\ \mu_{t}=C_{\mu }\rho_{W}\frac{k_{w}^{2}}{\varepsilon_{w}} \end{array}\right.$$

where *µ_t_* is the turbulent viscosity, *C_µ_* is the turbulent constant, *k_w_* is the turbulent kinetic energy, and *ε_w_* is the turbulent dissipation rate. However, only hydrogen gas flows in the cathode. Therefore, the single-phase flow model is applied to calculate the flow in the cathode. The continuity and momentum equations are used to calculate the flow. It is expressed as follows [26]:

(10) $$\left\{ \begin{array}{l} \nabla \cdot \left(\rho U\right)=0\\ \nabla \cdot (\rho UU)=-\nabla P+\nabla \cdot (\mu \nabla U)-\frac{\mu U}{\kappa_{D}} \end{array}\right.$$

where *ρ* and *µ* are the density and viscosity (listed in Table 3), respectively, of the fluid, *U* and *p* represent the velocity and pressure, respectively, of hydrogen, and *κ_D_* is the permeability of the PTL. It is calculated by the Carman-Kozeny equation, which is expressed as follows [27]:

(11) $$\kappa_{D}=\frac{d_{f}^{2}\varepsilon^{3}}{16k_{ck}{\left(1-\varepsilon \right)}^{2}}$$

where *d_f_* is the fiber diameter and *ε* is the porosity of the PTL. *k_ck_* (=4.28) is the Carman-Kozeny constant. 

### 2.3. Boundary and Initial Conditions

To couple the electrochemical reaction and two-phase flow in the anode, it is necessary to verify the boundary condition between the anode’s PTL and membrane. The volume fluxes of oxygen and water are specified as functions of the current density and are expressed as follows [18]:

(12) $$\left\{ \begin{array}{l} {\dot{V}}_{o}\cdot \vec{n}=\frac{M_{O}i}{4F\rho_{O}}\\ {\dot{V}}_{W}\cdot \vec{n}=-\frac{M_{W}i}{2F\rho_{W}} \end{array}\right.$$

where $\dot{V_{O}}$ and $\dot{V_{W}}$ are the volume fluxes of oxygen and water, respectively, since the reaction, $\vec{n}$ is the normal vector of the membrane boundary, and *M_O_* and *M_W_* are the molecular weights of oxygen and water, respectively. The volume fraction at the boundary layer of the membrane is specified by the volume flux of oxygen and water. To calculate the volume fraction at the boundary, we apply the mass conservation equation at the boundary layer near the membrane. It is expressed as follows:

(13) $$\alpha_{W,elect}=\frac{\alpha_{W}+{\dot{V}}_{O}}{1+({\dot{V}}_{W}+{\dot{V}}_{O})}$$

where *α_W,elect_* is the volume fraction of water at the interface between the membrane and PTL after the reaction.

Liquid water flows into the PEMWE’s anode, and the oxygen-water mixture flows out of it. Hydrogen is generated by an electrochemical reaction in the membrane, and only hydrogen flows out of the cathode. The temperature at the inlet is 80 °C. The boundary conditions at the inlet and outlet are calculated by:

(14) $$\left\{ \begin{array}{l} U=Q/A_{in}\\ P_{out}=P_{0} \end{array}\right.\begin{array}{c} \left(inlet\right)\\ \left(outlet\right) \end{array}$$

where *Q* is the flow rate (listed in Table 4) and *A_in_* is the inlet area (listed in Table 1). The simulations were conducted using OpenFOAM^®^. 

### 2.4. Experimental Setup

**Figure 2:**
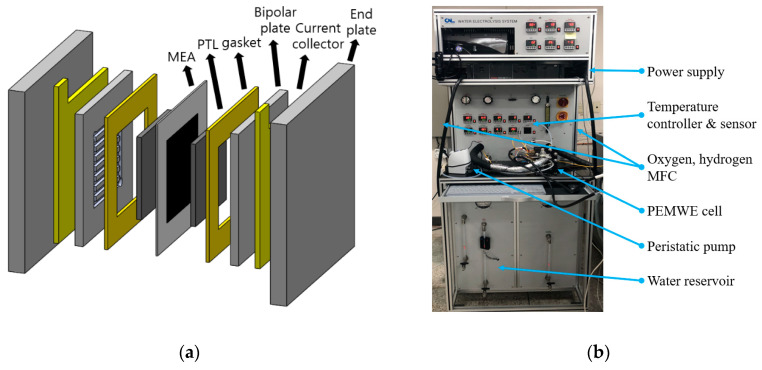
(**a**) Diagram of PEMWE and (**b**) experimental equipment manufactured by the CNL Energy Corp.

The experimental equipment for the model validation was configured as follows. As shown in Figure 2a, a Nafion™ (Wilmington, DE, USA) 115 membrane, with an active area of 50 mm × 50 mm, was used to separate the oxygen and hydrogen. At the anode, a titanium electrode was used for the PTL, and an Ir–Pt catalyst was used for the OOR [28]. At the cathode, a carbon paper was used for the PTL, and a Pt catalyst was used for the HRR. The experimental equipment manufactured by CNL Energy Corp. (Seoul, Korea) was used (Figure 2b) to measure the performance of the PEMWE. For the experiment, the flow rate of water, outlet pressure, and inlet temperature were set as 20 cm^3^/min, 101,325 Pa, and 80 °C, respectively.

## 3. Results and Discussion

### 3.1. Model Validation

**Figure 3:**
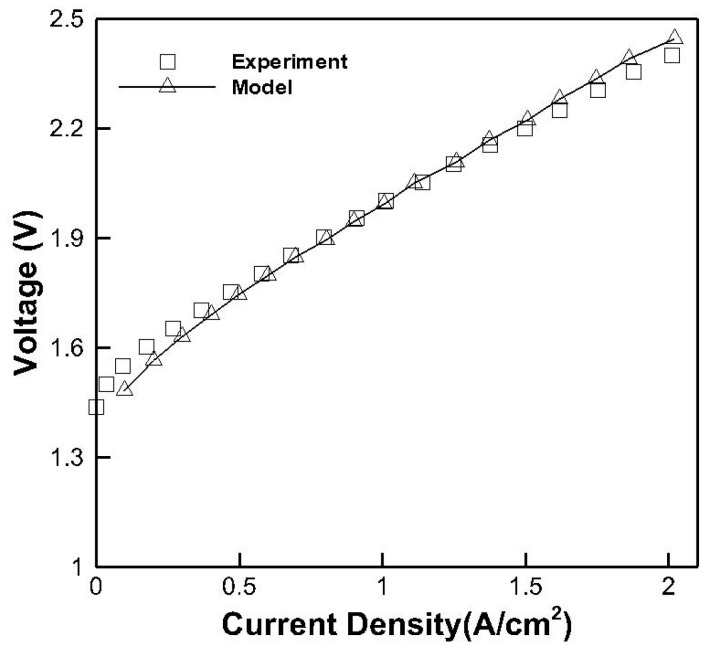
Model validation performed at a flow rate of 20 cm^3^/min.

The variation in the operating voltage according to the current density was observed for the experimental data and numerical model. The results calculated by the numerical model were validated by the experimental results shown in Figure 3. When the PEMWE is operated at a low current density, the operating voltage increases by the activation overpotential. The overpotential decreases as the amount of catalyst in the membrane increases. In this range, the operating voltage of the numerical model is lower than that for the experimental data, and the maximum error is 4.38%. When the PEMWE is operated at the mid-level current density, the operating voltage increases by the ohmic loss. The slope of the i-v curve increases with the increasing internal resistance of the cell. In this range, the slope of the numerical model is higher than that for the experimental data, and the maximum error is 0.26%. When the PEMWE is operated at a high current density, the operating voltage increases by the mass transfer. The operating voltage increases as the reaction product accumulates in the cell. The maximum error in this range is 1.8% and average error of the numerical model is 1.07%, and the residuals of the numerical model were converged under 5 × 10^−9^. Based on the results, we concluded that this numerical model is sufficient to explain the experimental data.

### 3.2. Analysis of Electrochemical Reaction of PEMWE

**Figure 4:**
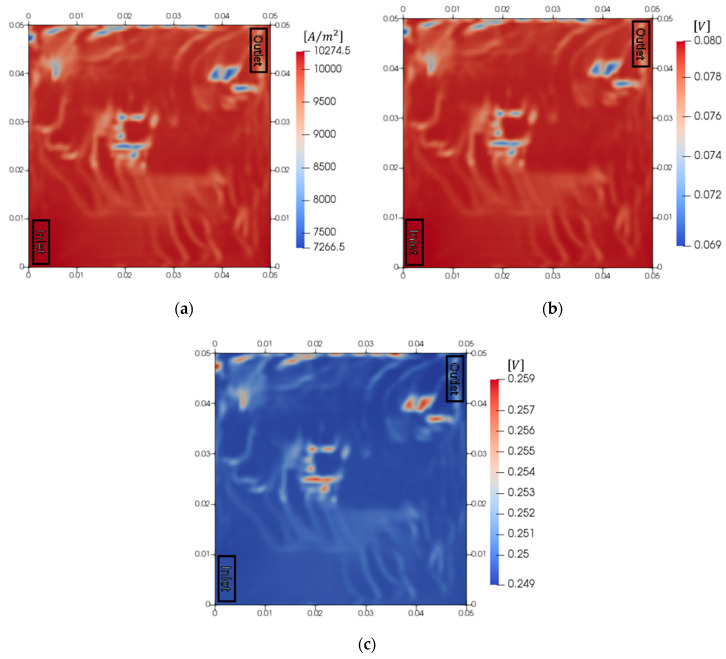
Electrochemical behaviors in PEMWE at a current density of 10,000 A/m^2^ (time = 1 s). (**a**) Current density, (**b**) overpotential of cathode reaction and (**c**) overpotential of anode reaction.

Figure 4 presents the results of the electrochemical reaction in the five-serpentine flow field. The flow rate of the PEMWE was 20 cm^3^/min, and the current density was 10,000 A/m^2^. The current density distribution and overpotentials of the anode and cathode were analyzed at time = 1 s. The maximum current density (10,300 A/m^2^) was observed near the inlet, and the minimum current density (7300 A/m^2^) was observed at the middle of the membrane and near the outlet. The current density at a certain location near the middle of the membrane and outlet decreased abruptly. This was a result of a decrease in the active area for the reaction, due to a disturbance in the catalyst-water interface, which, in turn, was caused by the generated oxygen bubbles. In the cathode, the influence of the oxygen bubbles was negligible since the cathode contained only hydrogen. Therefore, the overpotential of the cathode reaction was high where the local current density was high. The maximum overpotential of the cathode (0.080 V) was observed near the inlet, and the minimum overpotential (90.069 V) was observed at the location where the current density was low. The average overpotential was 0.079 V. Meanwhile, the oxygen bubbles that accumulated in the anode disturbed the contact between the water and catalyst. This decreased the active area for the reaction, which, in turn, caused the reaction overpotential to increase. The maximum overpotential in the anode (0.259 V) was observed at the location where the current density was low, and the minimum overpotential (0.249 V) was observed near the inlet. The average overpotential was 0.250 V. The overpotential of the cathode was lower than that of the anode (therefore, only the anode behaviors were analyzed subsequently). As a result, the performance decreased where the oxygen gas accumulated. Therefore, it is important to emit the generated oxygen gas to increase the performance of the PEMWE.

**Figure 5:**
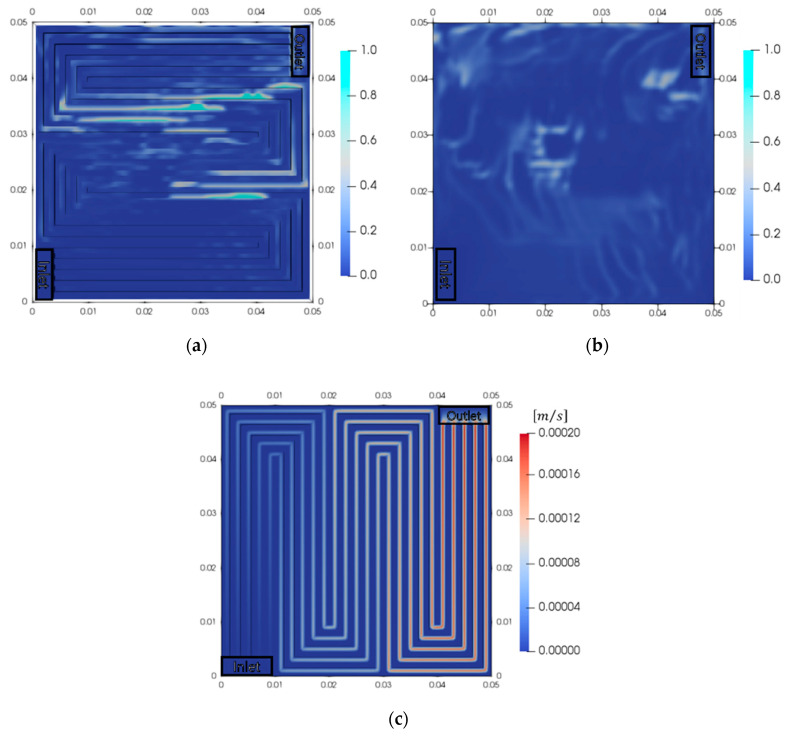
Fluid distributions in anode and cathode at a current density of 10,000 A/m^2^. (**a**) Volume fraction of oxygen in the channel, (**b**) volume fraction in the porous transport layer (PTL), and (**c**) hydrogen velocity in the cathode.

The decrease in performance was determined by the amounts of oxygen bubbles and the reactant. Therefore, the volume fractions of oxygen in the anode and the hydrogen velocity in the cathode were analyzed (Figure 5). Oxygen gas flows in the order of the membrane, PTL, and channel by convection. When oxygen gas is accumulated in the channel, the volume fraction of oxygen in PTL increases due to the disturbance of flow by convection. The maximum volume fraction in the channel was 0.993, which implied that that the removal of oxygen gas from the PTL was not smooth. The oxygen bubbles that accumulated hindered the flow of the oxygen generated in the PTL to the channel. Therefore, the active area of the catalyst decreased. The location where the oxygen accumulated in the PTL (shown in Figure 5b) was identical to that where the current density was low (shown in Figure 4a). The oxygen bubbles disturbed the contact between the catalyst and water, thereby, decreasing the active area for the reaction. This, in turn, decreased the performance of the PEMWE. However, the velocity of hydrogen increased as it flowed through the channel. The maximum velocity of hydrogen (1.96 × 10^−4^ m/s) was observed near the outlet. Only hydrogen was present in the cathode, and hydrogen exerted a negligible influence on the performance. Therefore, the removal of oxygen is important for increasing the performance of the PEMWE.

### 3.3. Analysis of Electrochemical Reaction According to the Current Density

**Figure 6:**
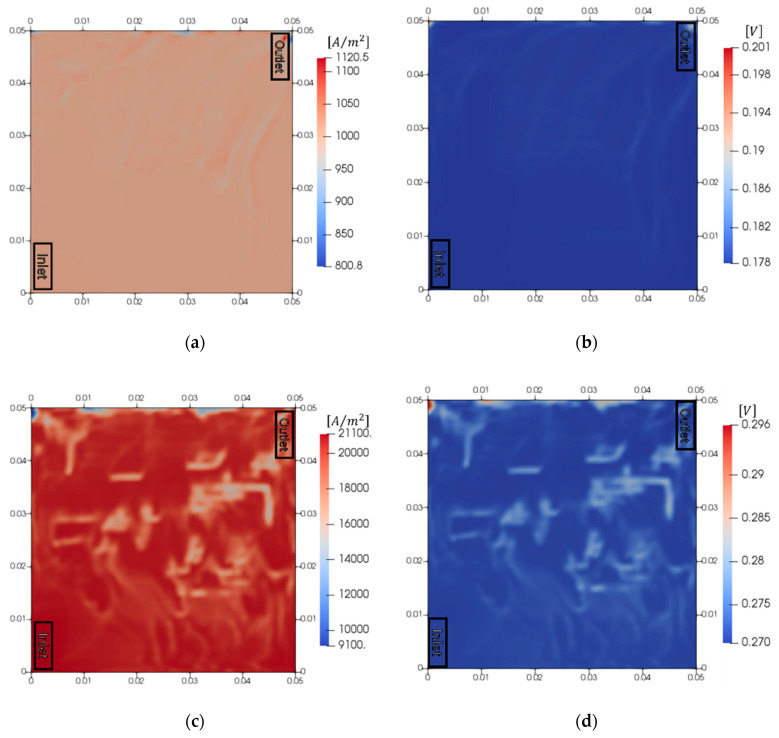
Electrochemical behaviors according to the current density. (**a**) Current density distribution and (**b**) overpotential at a low current density (1000 A/m^2^), (**c**) current density distribution and (**d**) overpotential at a high current density (20,000 A/m^2^).

Figure 6 shows the result of the electrochemical reaction with respect to the current density. We set the low and high current densities as 1000 and 20,000 A/m^2^, respectively. The flow rate of water was 20 cm^3^/min. The current density and overpotential of the anode were analyzed. When the PEMWE operated at a low current density, the current density distribution became more uniform than that for the operating current density of 10,000 A/m^2^. The maximum and minimum current densities were 112.0% and 80.0%, respectively. This indicates that the oxygen bubbles caused a smaller decrease in performance at a low current density. Moreover, the maldistribution of overpotential in this case was low. However, the maldistribution of current density intensified when the PEMWE operated at a high current density. The maximum and minimum current densities were 211.0% and 90.1%, respectively, which amounts to a difference of 12,000 A/m^2^. The maldistribution of the current density was observed at a high current density. However, the current density distribution was more uniform when it was low, which indicated that the maldistribution intensifies at a high current density. Therefore, it is important to eliminate the maldistribution of the current density to increase the performance of the PEMWE when it is operated at a high current density.

**Figure 7:**
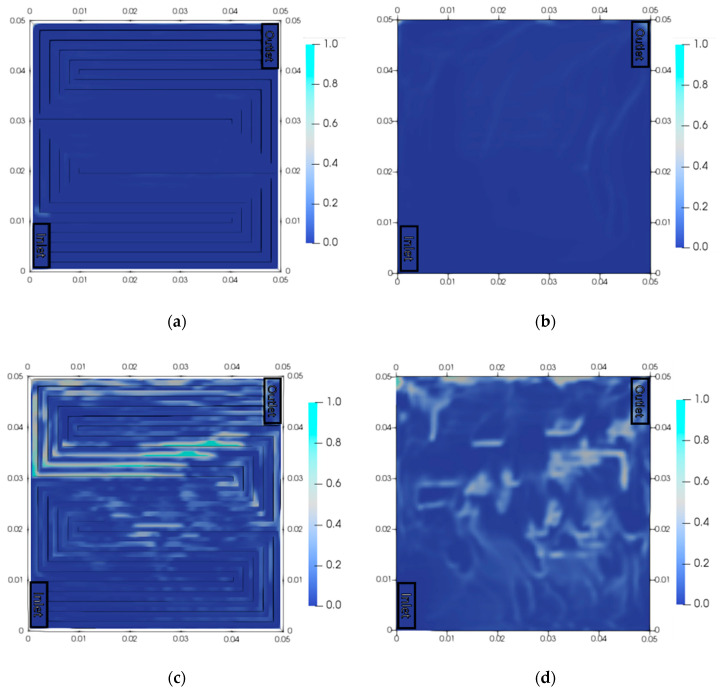
Volume fraction of oxygen (**a**) in the channel and (**b**) in the PTL at 1000 A/m^2^, and (**c**) in the channel and (**d**) in the PTL at 20,000 A/m^2^.

Figure 7 shows the volume fraction of oxygen in the channel and that in the PTL. The maldistribution of oxygen bubbles was marginal at 1000 A/m^2^. The maximum volume fraction of oxygen (0.620) was observed at the outlet. Most of the oxygen gas was emitted from the PEMWE at a low current density. Therefore, the maximum volume fraction in the PTL and channel was 0.166. However, the maldistribution of oxygen was observed at 20,000 A/m^2^. The maximum volume fraction of oxygen (0.977) was observed in the channel. A large quantity of oxygen gas accumulated in the elbow part of the channel, and oxygen bubbles were distributed across the channel. Although a large amount of oxygen is generated by the reaction, it was difficult for the oxygen in PTL to flow, since there was already oxygen in the channel. Therefore, the maldistribution of oxygen intensified, then the operating current density was at 10,000 A/m^2^. The influence of oxygen bubbles was higher at a higher current density, and the decrease in performance was larger. Therefore, the removal of oxygen is important when the PEMWE is operated at a high current density. Furthermore, it is necessary to compare the performances of various channel designs to distribute the product uniformly and resolve the maldistribution.

### 3.4. Performance Analysis of Serpentine and Parallel Channel for PEMWE

**Figure 8:**
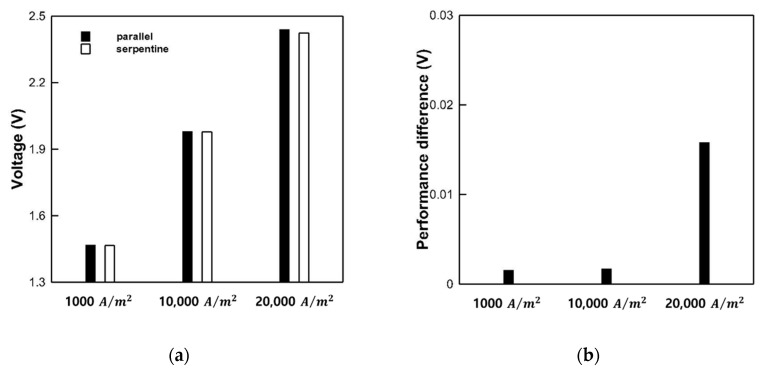
Performance of parallel and serpentine flow fields at 1000, 10,000, and 20,000 A/m^2^: (**a**) Operating voltage according to the current density and (**b**) performance difference according to the flow field.

Next, we compared the performances of the parallel and serpentine flow fields (shown in Figure 8). We set the current density to 1000 A/m^2^ (low), 10,000 A/m^2^ (mid-level), and 20,000 A/m^2^ (high). At 1000 A/m^2^, the operating voltages of the parallel and serpentine flow fields were 1.469 and 1.467 V, respectively. The difference between the parallel and serpentine flow fields was marginal, since most of the oxygen gas was emitted. Therefore, the influence of oxygen was also low. At 10,000 A/m^2^, the operating voltages of the parallel and serpentine flow fields were 1.979 and 1.978 V, respectively. The difference between the two flow fields was marginal in this case, as well. However, the difference was large at 20,000 A/m^2^. The operating voltages of the parallel and serpentine flow fields were 2.439 and 2.423 V, amounting to a difference of 0.016 V. In the serpentine flow field, the flow resistance of the fluid in the channel was high due to the complex shape of the field designed. Thereby, water collected in the channel. Then, the generated oxygen gas flowed through the PTL due to the surface tension of the water. Therefore, it is advantageous to remove the oxygen gas in the case of the serpentine flow field, unlike the parallel flow field. Based on the results, the performance of the serpentine flow field is higher than that of the parallel flow field for the PEMWE.

## 4. Conclusions

In this study, a transient model was developed to analyze the two-phase flow and electrochemical behaviors in the PEMWE. We analyzed the performance of the five-serpentine flow field. Then, we analyzed the performance of the PEMWE at various current densities. Finally, the performances of the serpentine and parallel channel designs were analyzed. The major observations can be summarized as follows:

1. The electrochemical behaviors and fluid flow were analyzed. A maldistribution of the current density was observed where the oxygen gas had accumulated in the PTL. The difference in the current density in the cell was a maximum of 3000 A/m^2^ at an operating current density of 10,000 A/m^2^. The maximum volume fraction in the PTL was 0.618, which is higher than the average volume fraction by 0.580. When oxygen accumulated in the PTL, the active area for the reaction decreased, since the contact between the water and catalyst was disturbed. This caused a decrease in the performance of the PEMWE. Hence, the removal of oxygen gas is important for increasing the performance of the PEMWE.

2. The performance of the PEMWE was analyzed according to the current density. We set low and high current densities as 1000 and 20,000 A/m^2^, respectively. At an operating current density of 1000 A/m^2^, the maximum current density was 112.0%. This implied that the generated oxygen was removed smoothly at a low current density. However, a maldistribution of the current density was observed at an operating current density of 20,000 A/m^2^; the corresponding maximum current density was 211.0%. Simultaneously, a maldistribution of the volume fraction of oxygen was observed, and the maximum volume fraction was 0.977. Therefore, the performance of the PEMWE decreased owing to the presence of oxygen bubbles at the high current density. Hence, the removal of oxygen gas is important when PEMWE is operated at a high current density.

3. We analyzed the performance of parallel and serpentine flow fields at current densities of 1000, 10,000, and 20,000 A/m^2^. At the low current densities of 1000 and 10,000 A/m^2^, the difference in performance between the flow fields was low. However, the operating voltage of the serpentine flow field was 0.016 V lower than that of the parallel flow field at the high current density of 20,000 A/m^2^. In the serpentine flow field, the accumulated water in the channel disturbed the flow of oxygen to the channel. Therefore, the generated oxygen gas flowed through the PTL. Therefore, compared to the parallel flow field, the serpentine flow field has an advantage with respect to the removal of oxygen gas. Therefore, we conclude that the performance of the serpentine flow field is higher than that of the parallel flow field.

## Figures and Tables

**Table 1:** Geometric parameters of PEMWE.

Parameter	Symbol	Value
Area of electrolyzer	A_cell_	25 cm^2^
Area of inlet and outlet	A_in_, A_out_	27 mm^2^
Membrane thickness	L_m_	0.13 mm
PTL thickness at anode	L_A_	0.36 mm
PTL thickness at cathode	L_C_	0.20 mm
Channel height	H_C_	1 mm
Channel width	W_C_	1 mm
Lib width	W_L_	1 mm

**Table 2:** Parameters of the electrochemical reaction.

Parameter	Symbol	Value
Reference exchange current density for ORR	*i_A_*_0_ *^ref^*	1 × 10^−3^ A/m^2^ [12]
Reference exchange current density for HOR	*i_C_*_0_ *^ref^*	1 A/m^2^ [9]
Effective activation energy for anode reaction	*E_A_*	18 × 10^3^ J/mol [10]
Effective activation energy for cathode reaction	*E_C_*	76 × 10^3^ J/mol [10]
Transfer coefficient for anode reaction	*α_A_*	0.5
Transfer coefficient for cathode reaction	*α_C_*	0.5
Conductivity of membrane	*σ*	10 S/cm
Roughness factor of electrode	*γ_m_*	100 [23]
Equilibrium constant of reaction	*K_A,C_*	6.2 [10]

**Table 3:** Properties of the fluid at 80 °C.

Parameter	Symbol	Value
Density of liquid water	*ρ_W_*	971.60 kg/m^3^
Density of oxygen	*ρ_O_*	1.089 kg/m^3^
Density of hydrogen	*ρ_H_*	0.0686 kg/m^3^
Viscosity of liquid water	*µ_W_*	0.355 kg/ms
Viscosity of oxygen	*µ_O_*	2.341 × 10^−5^ kg/ms
Viscosity of hydrogen	*µ_H_*	9.932 × 10^−6^ kg/ms

**Table 4:** Initial and boundary conditions of the PEMWE operation.

Parameter	Symbol	Value
Flow rate of water	*Q_A_*	20 cm^3^/min
Inlet temperature	*T_in_*	80 °C
Outlet pressure	*P_O_*	101,325 Pa

## Nomenclature

*A*	Area of inlet, outlet of electrolyzer (m^2^)
*ASR*	Area specific resistance (Ω m^2^)
*E*	Voltage (V), activation energy (J/mol)
*F*	Faraday’s constant (C/mol)
*H*	Height of channel (m)
*i*	Current density (A/m^2^)
*i*_0_	Exchange current density (A/m^2^)
*L*	Thickness of membrane and PTL (m)
*P*	Pressure (Pa)
*Q*	Flow rate (cm^3^/min)
*R*	Gas constant (J/mol K)
*U*	Velocity of fluid (m/s)
*T*	Temperature (K)
$\dot{V}$	Volume flux of oxygen and water (m^3^/s)
*W*	Width of channel and lib (m)
*α*	Transfer coefficient, volume fraction of fluid
*η*	Overpotential (V)
*µ*	Viscosity of fluid (kg/m s)
*ρ*	Density of fluid (kg/m^3^)
*σ*	Conductivity of membrane (S/m)
*γ*	Roughness factor of electrode
*Subscripts*	
*A*	Anode
*C*	Cathode
*H*	Hydrogen
*i*	Pertaining to species i
mem	Membrane
O	Oxygen
ohm	Ohmic loss
W	Water
*Acronyms*	
PEMWE	Polymer electrolyte membrane water electrolyzer
MEA	Membrane electrode assembly
PTL	Porous transport layer
OCV	Open circuit voltage
ORR	Oxygen reduction reaction
OOR	Oxygen oxidation reaction
HRR	Hydrogen reduction reaction
HOR	Hydrogen oxidation reaction
